# Colon Adenoma After Diagnosis of Immune Checkpoint Inhibitor-mediated Colitis

**DOI:** 10.7150/jca.86635

**Published:** 2023-08-28

**Authors:** Antonio Pizuorno Machado, Malek Shatila, Enrico N. De Toni, Helga-Paula Török, Jessica Philpott, Dan Zhao, Yan Zhou, Krishnavathana Varatharajalu, Mehnaz A. Shafi, Hao Chi Zhang, Anusha S. Thomas, Yinghong Wang

**Affiliations:** 1 Department of Internal Medicine, The University of Texas Health Science Center, Houston, TX, USA.; 2 Department of Gastroenterology, Hepatology, and Nutrition, The University of Texas MD Anderson Cancer Center, Houston, TX, USA.; 3 Department of Medicine II, University Hospital, Ludwig-Maximilians-University Munich, Munich, Germany.; 4 Inflammatory Bowel Disease Center, Cleveland Clinic, Cleveland, OH, USA.; 5 Department of Gastrointestinal Medical Oncology, The University of Texas MD Anderson Cancer Center, Houston, TX, USA.; 6 Department of Hospital Medicine, The University of Texas MD Anderson Cancer Center, Houston, TX, USA.

**Keywords:** immune checkpoint inhibitor, colitis, adenoma, colonoscopy, surveillance

## Abstract

**Purpose:** While the occurrence of colitis during immune checkpoint inhibitor (ICI) treatment is recognized as a sign of robust immune activation and correlates with better oncological outcomes, the long-term impact of ICI-mediated colitis on the colonic mucosa has not been studied. We thus aim to describe the colonoscopy and histology findings in patients at a follow-up time of ≥ 6 months post initial colitis event.

**Methods:** This retrospective analysis included adult cancer patients diagnosed with ICI colitis at a tertiary cancer center between October 2013 and June 2020. The study group included patients diagnosed with immune mediated colitis who had also undergone a follow up colonoscopy or flex sigmoidoscopy. The control group was patients exposed to ICI without immune mediated colitis. We reported patients' colitis clinical course, treatment, outcomes, and endoscopic and histologic features at diagnosis and at follow-up time of ≥ 6 months.

**Results:** Total 39 patients met the study criteria, with 82% being male, and 35.8% having melanoma. Most patients received a combination of CTLA-4 and PD-1/L1 inhibitors (82%). On initial endoscopic evaluation, inflammation without ulceration was reported in 76.9% of patients and active inflammation on histologic examination in 79.3% of patients. Most patients (79.4%) received corticosteroids, and 56.4% received add-on selective immunosuppressive therapy. Four patients received fecal microbiota transplantation. On follow-up, new incidence of colonic polyps was reported in 51.2% of patients, including adenomas in 33.3% among the colitis patients with median follow up duration of 12 months. The incidence of adenoma polyps 12 months after the colitis event was significantly higher compared to the control group without colitis based on the time-to-event analysis (p=0.041).

**Conclusion:** At a median follow up of 12 months after their initial colitis diagnosis, 51.2% of the patients had new incidence of colonic polyps, including a third with adenoma, at a significantly higher incidence than the control group without colitis. Studies with larger sample sizes are needed to further define the long-term impact of colitis and its treatments on colon health and to refine recommendations for surveillance of colonic adenomas and colorectal cancer.

## Introduction

Immune checkpoint inhibitors (ICIs) have been increasingly used as a treatment for various cancers. These agents act by releasing the immune system from specific inhibitory checkpoints to trigger a tumor-specific immune-mediated cytotoxic effect 1. The best characterized targets of ICIs are cytotoxic T-lymphocyte-associated antigen 4 (CTLA-4), programmed cell death protein 1 (PD-1), and programmed cell death ligand 1 (PD-L1). Treatment with monoclonal antibodies targeting these proteins is associated with increased T-cell activation and effective anti-tumor immune responses. Yet, unleashing the immune system from negative regulation comes with well-known adverse reactions caused by loss of self-tolerance and excessive inflammatory activity, collectively known as immune-related adverse events 2.

Immune-mediated colitis, a common adverse reaction observed in patients undergoing treatment with ICIs, has been extensively studied over the past 5 years 3-6. This condition requires exclusion of other alternative diagnosis as it frequently overlapped with features of inflammatory bowel disease, infectious colitis and graft-versus-host disease 7-8. The initial treatment for moderate and severe colitis consists from dietary considerations to steroids, followed by selective immunosuppressive therapy (e.g., infliximab, vedolizumab) in refractory cases, and achieves response rates exceeding 80% 9-10. Ustekinumab, tofacitinib, extracorporeal photopheresis and fecal microbiota transplantation are also reported to be effective 7, 11-14, and their use for steroid-refractory cases is being assessed.

It has been shown that prolonged course of colitis (>3 months) is associated with better long-term cancer outcomes and survival 6, 15. However, there are indications that along with this beneficial effect on cancer-dependent prognosis, prolonged inflammation of the intestine following treatment with ICIs might also affect the long-term consequences on the health of colonic mucosa. This is exemplified by the fact that colitis can persist for years even after ICI cessation 16. Therefore, we sought to elucidate the long-term outcome of immune-mediated colitis, in particular, the incidence of additional colon pathology.

## Methods

### Study design and population

This retrospective chart review is a descriptive, single-center study that included adult patients who were diagnosed with colitis after ICI treatment at a tertiary cancer center between October, 2013, and June, 2020. This study was approved by the institutional review board with a waiver of patients' informed consent. We identified patients 18 years or older who (1) were treated with ICIs for various types of cancer, (2) had a diagnosis of immune-mediated colitis, and (3) had colonoscopy/flex sigmoidoscopy at the time of colitis diagnosis and at follow-up at least 6 months later. Our control group included all patients that received ICI within the study window (1) that underwent serial colonoscopy or flexible sigmoidoscopy after initiating ICI, (2) had negative baseline scope, (3) without immune mediated colitis diagnosis. Patients with pre-existing inflammatory bowel disease (IBD), microscopic colitis, or other autoimmune gastrointestinal disorders were excluded.

### Clinical data

Demographic and cancer-related information such as age, gender, primary cancer type, stage, cancer treatments received and doses, and Charlson Comorbidity Index score were collected. Also collected were data related to the onset of colitis, such as date, cycles of ICI before colitis, type of ICI, and peak Common Terminology Criteria for Adverse Events (CTCAE) grades for colitis and diarrhea. Diagnosis of colitis was based on the clinical presentation and endoscopic and histologic features after the exclusion of other etiologies. Information about the treatment for colitis such as steroids, infliximab, and vedolizumab, including doses and start and end dates, was obtained as well. Colonoscopy/sigmoidoscopy and pathology findings at the time of colitis diagnosis and at follow-up were collected based on previously established characteristics 17-20; these included polyps, intervention (e.g., polypectomy, stricture dilation), and complications (e.g., bleeding, perforation). Polyp pathology was described as adenomatous or hyperplastic based on general terminology, given the lack of standardization on documentation for this population as what is well established in the IBD literature.

### Statistical analysis

The statistical analyses performed were descriptive in nature. The distributions of continuous variables were summarized by medians and interquartile ranges. The distributions of categorical variables were summarized by frequencies and percentages. These were calculated using SPSS 26. To calculate the incidence of adenoma, we included all cases from both colitis and control groups with negative colonoscopy at baseline who had a follow-up colonoscopy/flex sigmoidoscopy done at least 6 months but not more than 1 year later. Time to adenoma detection was modeled for 6 months to 1 year after baseline colonoscopy using the Kaplan-Meier method. Survival curves were compared using the log-rank test.

## Results

### Patient population, characteristics, and oncologic history

**Figure 1:**
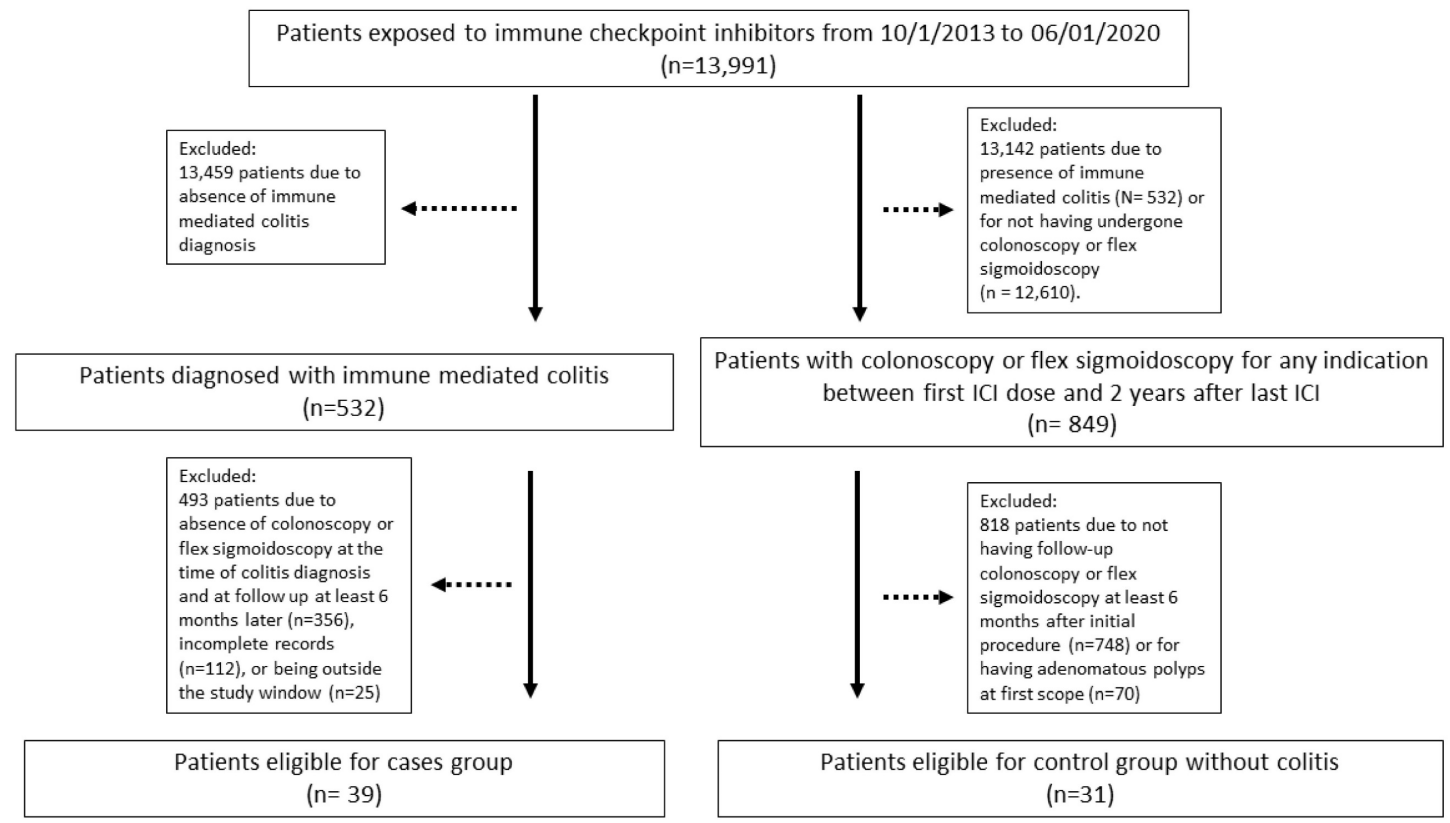
Patient selection flowchart.

Thirty-nine patients met the inclusion criteria for the colitis group (patient selection flowchart shown in **Figure 1**). Their characteristics are summarized in **Table 1**. The most common cancer was melanoma, followed by genitourinary cancer, gastrointestinal cancer, and lung/head and neck malignancies. Thirty-two patients (82%) received a combination of PD-1/L1 and CTLA-4 inhibitors, 5 patients (12.8%) received a PD-1/L1 inhibitor as monotherapy, while only 2 patients (5.1%) received a CTLA-4 inhibitor as monotherapy. Clinical response of colitis symptom was achieved in 33 patients (84.6%). Six patients (15.4%) had screening colonoscopy before their colitis diagnosis within a median duration of 10 months (**Table 3**), and all had detected polyps that were removed. In addition, 31 patients met the inclusion criteria for the control group for comparison of incidence of colon adenoma polyps (**Figure 1**).

### Endoscopic and histologic characteristics

At the time of colitis diagnosis, the most common endoscopic finding was non-ulcer inflammation in 30 patients (**Table 2**). In 7 patients, inflammation was evident only on histologic examination, with no obvious macroscopic changes of the colonic mucosa. Ulcers were found in only 2 patients. Histologic examination showed active inflammation in 31 patients. Polyps were found in 3 patients - 2 with hyperplastic polyps, 1 with adenoma, all of which were removed during the endoscopy.

On follow-up endoscopic examination, signs of persistent inflammation (erythema, erosions, vascular congestion) were still present in 16 patients (**Table 2**). In 20 patients (51.2%), sessile polyps were reported and removed. Thirteen of these patients had detection of new adenomatous polyps: 11 had tubular adenomas, and 2 had tubulovillous adenomas. Two patients had polyps smaller than 5 mm, and 11 patients had polyps larger than 5 mm. The majority of the polyps were located in the ascending colon in 4 patients (30.6%) followed by transverse colon in 3 patients (23%). The median duration was 7.1 months from colitis diagnosis to the first follow-up endoscopic examination and 12.6 months to the last follow-up endoscopic examination. The endoscopic and histologic characteristics by cancer type are summarized in **Supplemental Table 1**.

### Comparison of characteristics of patients with and without adenoma polyps

The median age of the 13 patients with adenoma polyps was 65 years (**Supplemental Table 2**), and the most common malignancy was melanoma. All the patients with adenoma received combination therapy with PD-1/L1 and CTLA-4 inhibitors. The endoscopic findings in most patients showed non-ulcer inflammation. In most patients, the distribution of inflammation and location of polyps was similar on follow-up endoscopy. Adenoma polyps were less likely to be found in patients who were receiving active colitis treatment at the time of follow-up endoscopy compared with patients who were not, despite similar ICI treatment status. Patients with adenoma polyps had a longer median duration between colitis onset and last follow-up endoscopy (14.8 vs 4.11 months, *p*=0.004). Otherwise, the characteristics were comparable between the patients with and without adenoma polyps.

**Figure 2:**
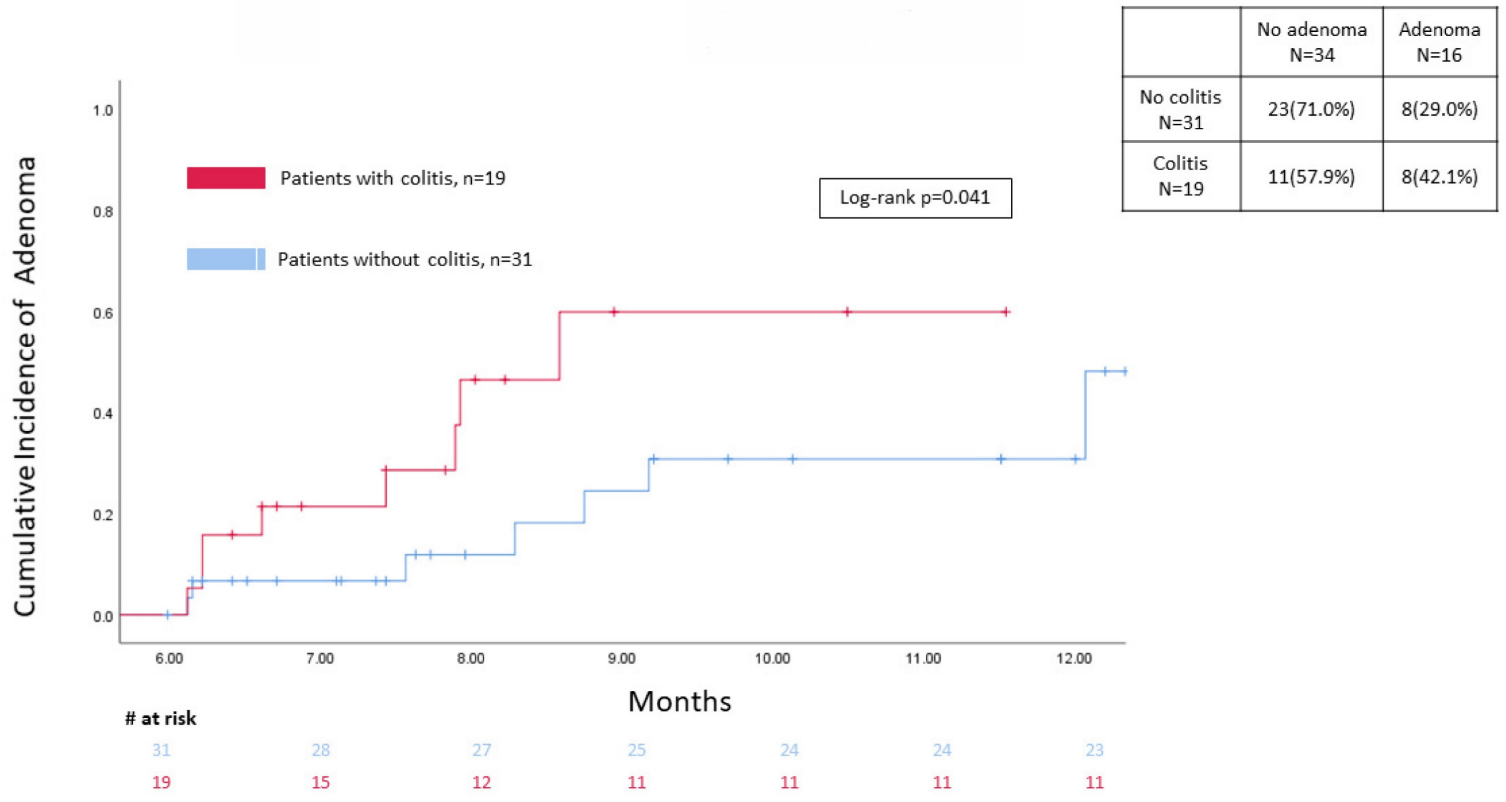
Kaplan-Meier for adenoma development between 6 months to 1 years of follow up.

A total of 849 patients in the control group had a colonoscopy/flex sigmoidoscopy done at baseline after ICI treatment, with only 31 patients having a follow-up colonoscopy/sigmoidoscopy within 6 months to 1 year afterwards. The incidence of adenoma at different time points between the two groups on follow-up endoscopy exam in the study window was presented in **Figure 2** showing that colitis patients had a significantly higher level on time-to-event analysis (*p*=0.041).

Univariate analysis (**Table 3**) identified longer duration from colitis onset to follow-up endoscopy as the only risk factor associated with adenoma. Age, cancer type, initial colonoscopy and histology findings, or colitis treatments were not associated with colon adenoma.

## Discussion

The long-term outcome of ICI colitis after medical treatment has not been well studied. Our data found that colon adenomatous polyps were newly reported in 33.3% of patients on follow-up endoscopic evaluation around 12 months after colitis. Furthermore, 41% of our patients had persistent histologic inflammation. With the concerning rate of new polyp detection within the short 12 months window following a colitis event, it is critical to develop the optimal surveillance program for colon adenoma and thereby reduce the risk of colon dysplasia and/or colon cancer.

Colon cancer is the third most common malignancy and the second most deadly cancer in the US 21. Certain chronic conditions, such as IBD, can increase the risk of colon cancer 22 secondary to persistent inflammation, long disease duration, extensive colitis distribution and higher severity, coexistent primary sclerosing cholangitis, and a family history of colon cancer 23 etc. In IBD patients, a histologic diagnosis of dysplasia (equivalent term for adenoma without IBD background) as well as serrated polyps carries a higher risk of developing more advanced neoplasia 24-25. Different advanced techniques (such as chromoendoscopy) are recommended to improve the early and efficacious detection of dysplasia and cancer. Many international societies recommend a first surveillance colonoscopy 8-10 years after diagnosis with extensive colitis followed by subsequent surveillance on an interval determined by various risk factors 26-29. Patients with primary sclerosing cholangitis specifically have a higher risk and require annual colonoscopy surveillance. Nonetheless common genetic and signaling pathways such as p53 should also be considered in the colitis associated cancer spectrum, inflammation can induce the production of pro inflammatory cytokines that can induce mutation in oncogenes such as p53, APC, K-ras and genomic instability by different mechanism promoting tumor proliferation and antiapoptotic properties or premalignant cells 30. IBD and ICI colitis share multiple similitudes, such as the disruption in immune surveillance pathways and acute and/or chronic gastrointestinal inflammation. Especially concerning is the recently reported study showing the prolonged colitis disease course for years after ICI termination, with or without overt clinical symptoms 10. Therefore, the elevated risk of colon cancer associated with IBD may also be anticipated and should be investigated in patients with ICI colitis. On the other hand, patients with mild ICI colitis on endoscopy evaluation, with shorter colitis course and minimal requirement of aggressive treatment, are usually considered a low-risk group. However, in our analysis, 7% of patients with normal endoscopy findings at colitis onset still experienced de novo colon adenomas later on, suggesting that even low-grade colonic inflammation may contribute to the pathogenesis of colon adenoma/cancer.

Available data have shown that early infliximab and vedolizumab treatment for moderate to severe immune-mediated colitis was associated with better colitis disease course and improved overall survival 10. The decision to start infliximab and vedolizumab was frequently based on the CTCAE grade of diarrhea/colitis as well as high-risk endoscopic features that were previously described 6. However, the safety of long-term use of potent immunosuppressants in patients with immune-mediated colitis remains unclear. Notably, a small increased risk of secondary malignancies such as lymphoma 31, skin cancers 32, and leukemia 33 was reported in IBD and rheumatological disease among patients on long term use of immunosuppressants who otherwise had no active malignancy. In particular, as the increasing volume of ICI-treated patients with chronic colitis with prolonged disease course, long-term maintenance with immunosuppressants has become more widely indicated and practiced. A recent study showed that the use of steroids and infliximab may impair overall survival among cancer patients 34. This brought to providers' attention the potential negative impact of immunosuppressants on cancer-related outcomes, which has not been extensively studied previously. With all these risk factors, patients with immune-mediated colitis could be predisposed to colon adenoma/cancer, which can be monitored and prevented by implementing an optimal surveillance strategy via colonoscopy to remove dysplastic lesions early on.

The progression from small adenomatous polyps to sporadic colorectal cancers usually take years' duration 35. Colonoscopy surveillance is associated with a substantially reduced incidence of colorectal cancer and its associated mortality, therefore, it has become the standard clinical practice for the general population 36. Different characteristics and number of advanced adenomatous lesions may require more intense colonoscopy surveillance programs to maximize its benefit of cancer prevention, and thus screening intervals range from 3 to 10 years 37. The surveillance for patients with existing colon cancer history is even more intensified 38. In our cohort, only 6 patients (15.3%) had undergone screening colonoscopy before their colitis episode, a finding that could reiterate the need for up-to-date surveillance colonoscopy among cancer population. Despite the lack of high-quality data, multiple retrospective studies have observed a higher incidence of colon adenoma/cancer among cancer populations regardless of colon cancer history 39-42. This association may further contribute to the outcome of advanced adenomatous lesions in the colon that we observed within a short time after the diagnosis of immune-mediated colitis in the setting of active malignancy and ICI treatment. Another confounding factor that should be considered is that the low rate of adenoma detection or reporting at the time of colitis diagnosis could be due to the masking effect from significant mucosal inflammation and higher threshold for endoscopists to perform polypectomy owing to concerns of bleeding, perforation, and poor healing 43. This could also contribute to the rapid detection of adenoma just 6 months after colitis diagnosis following the initial scope evaluation. In addition, patients with colitis may have suboptimal bowel preparation for the endoscopy (up to 40% of cases in our data), which could impair the visualization of polyps.

Although this is the largest study on this topic to our knowledge, it has limitations. First, it is a single-center retrospective review. Second, the small sample size limits the power of subgroup analysis to compare different risk factors. Third, the primary cancer outcomes could be affected by multiple factors that limit the follow-up duration and the feasibility of repetitive colonoscopy, leading to a patient selection bias and different risk levels of second malignancy. Fourth, the lack of a standardized surveillance program after a colitis event can limit the risk assessment and stratification of subsequent management of colon health and colon cancer prevention.

In our study, more than 50% of patients diagnosed with immune-mediated colitis had de novo polyps reported on follow-up colonoscopy/flex sigmoidoscopy 12 months after the colitis diagnosis, and more than half the lesions were adenomatous, which is above the incidence of general patients without colitis history. This should raise concerns of rapid development and detection of colon adenoma after colitis diagnosis that is notably faster than in the general cancer population without colitis. To maximize the reduction of secondary colon cancer risk in this population, a prompt surveillance colonoscopy at 1 year after colitis diagnosis should be considered. Further assessment of this risk to determine the optimal interval for subsequent surveillance is warranted.

## Supplementary Material

Supplementary tables.

## Figures and Tables

**Table 1:** Patient Characteristics, N=39

Characteristic	No (%)
Age, years, median (IQR)	64.2 (49-69.8)
Sex, male*	32 (82)
Race, white†	35 (89.7)
Cancer type	
Melanoma	14 (35.8)
Genitourinary	10 (25.6)
Lung/head and neck	6 (15.3)
Gastrointestinal	7 (17.9)
Hematological	1 (2.5)
Renal	1 (2.4)
Type of ICI	
Anti-CTLA-4 monotherapy	2 (5.1)
Anti-PD-1/L1 monotherapy	5 (12.8)
Combination anti-CTLA-4 and anti-PD-1/L1	32 (82)
Duration of follow-up from colitis diagnosis to last follow-up, months, median (IQR)	14.2 (9.1-41.2)

Abbreviations: CTLA-4, cytotoxic T lymphocyte antigen 4; ICI, immune checkpoint inhibitor; IQR, interquartile range; PD-1/PD-L1, programmed cell death 1/programmed death ligand 1. *7 patients (18%) were females. †3 patients (7.6%) were Hispanic and 1 patient (2.5%) African American.

**Table 2:** Endoscopy-related characteristics, N=39

At the time of colitis diagnosis	No. (%)
Endoscopic findings	
Inflammation	
Ulcers	2 (5.1)
Non-ulcer inflammation	30 (76.9)
Normal	7 (17.9)
Other findings	
Polyps ¶	3 (7.6)
Histologic findings	
Acute inflammation	18 (46.1)
Chronic inflammation	10 (25.6)
Microscopic colitis	3 (7.6)
Normal	8 (20.5)
Treatment of IMC	
Corticosteroids only	16 (41.0)
Corticosteroids plus infliximab only	8 (20.5)
Corticosteroids plus vedolizumab only	7 (17.9)
Multiple treatment lines‡	8 (20.5)
Colitis maintenance treatment (> 3 doses of accumulative biologic agents: infliximab, vedolizumab, ustekinumab)	8 (20.5)
**At the time of follow-up endoscopy**	
Duration from colitis diagnosis to first follow-up endoscopy, months, median (IQR)	7.1 (2.9-21.7)
Duration from colitis diagnosis to last follow-up endoscopy, months, median (IQR)	12.6 (8.2-18.8)
On active colitis treatment	10 (25.6)
On active ICI treatment on follow-up endoscopy	6 (15.3)
Endoscopic findings	
Inflammation	
Erythema, erosions, vascular congestion and inflammation	16 (41)
Normal	23 (58.9)
Others	
New polyps	20 (51.2)
Stricture	0 (0)
Perforation	0 (0)
Histologic findings	
Inflammatory findings	
Inflammation†	19 (48.7)
Normal histologic findings	20 (51.2)
Others	
Adenoma polyps §	13 (33.3)
Endoscopic intervention or surgery	
Polypectomy (out of 20 patients with polyps)	20 (100%)
Stricture dilation	0 (0)
Surgical resection of colon cancer	0 (0)
All cause mortality	7 (21.9)

Abbreviations: CTCAE v5, Common Terminology Criteria for Adverse Events version 5; ICI, immune checkpoint inhibitor; IMC, immune-mediated colitis; IQR, interquartile range; TNF, tumor necrosis factor; FMT, fecal microbiota transplantation *The 3 patients with cancer on histologic examination on follow-up colonoscopy had a previous diagnosis of colon cancer. †Among patients with active histologic inflammation on follow-up: 4 patients (10.2%) had acute inflammation, 11 patients (28.2%) had chronic inflammation, 1 patient (2.5%) had acute and chronic inflammation, 3 patients (7.6%) had microscopic colitis. ¶ 2 patients had hyperplastic polyps and 1 patient had an adenoma. All of them were <5 mm in size and removed. These patients had non-ulcer inflammation at the time of colitis diagnosis. § 11 patients had tubular adenomas, 2 had tubulovillous adenomas. 1 patient had polyps <5 mm in size, and 11 had polyps >5 mm, and all were removed. ‡ 3 patients received both infliximab and vedolizumab, 1 patient received infliximab and ustekinumab, and 4 patients received FMT alongside other treatments (1 FMT + steroids, 1 FMT + infliximab + vedolizumab, 2 FMT + vedolizumab)

**Table 3:** Univariate analysis for colon adenoma, N=39

Variables	OR (CI)	*p*
Age	1.0 (0.9-1.1)	0.191
Cancer type (melanoma vs others)	1.9 (0.5-7.6)	0.348
Initial colitis endoscopic findings (active inflammation vs normal)	0.6 (0.1-3.2)	0.557
Initial colitis histologic findings (presence of inflammation vs normal)	0.8 (0.2-4.0)	0.779
Immunosuppressant treatment for initial colitis (biologics vs no biologics)	0.5 (0.1-2.1)	0.364
Active inflammation on follow-up endoscopy	0.3 (0.1-1.3)	0.120
Active inflammation on follow-up histology	0.4 (0.1-2.5)	0.355
Adequate or better preparation vs air or worse preparation for initial endoscopy	0.8 (0.2-3.9)	0.789
Duration of last colonoscopy screening before colitis onset to the last follow-up colonoscopy	1.1 (0.9-1.3)	0.442
Long term colitis maintenance with biologics vs no	1.3 (0.3-6.4)	0.779
Adequate or better preparation vs fair or worse preparation for follow-up endoscopy	0.4 (0.1-2.6)	0.341
Follow-up duration from colitis diagnosis to follow-up endoscopy	1.05 (1.00-1.11)	0.040
