# Supplementary material for: Colon Adenoma After Diagnosis of Immune Checkpoint Inhibitor-mediated Colitis
Source: J Cancer. 2023 Aug 28;14(14):2686–93. doi: 10.7150/jca.86635 (PMC10539391; doi:10.7150/jca.86635)
Supplement: Supplementary file 1 — Supplementary tables. [file jcav14p2686s1.pdf]

| **Supplemental Table 1**. Endoscopy-related characteristics by cancer type, N=39 | | | | | | | |
|---|---|---|---|---|---|---|---|
| | Total | Melanoma | GU | GI | Lung Head/Neck | Hematological | Renal |
| **At the time of colitis diagnosis** | **No. (%)** | 14 (35.8) | 10 (25.6) | 7 (17.9) | 6 (15.3) | 1 (2.5) | 1 (2.4) |
| Endoscopic findings | | | | | | | |
| Inflammation | | | | | | | |
| Ulcers | 2 (5.1) | 1 (7.1) | 1 (10) | 0 (0) | 0 (0) | 0 (0) | 0 (0) |
| Non-ulcer inflammation | 30 (76.9) | 12 (85.7) | 6 (60) | 4 (57.1) | 6 (100) | 1 (100) | 1 (100) |
| Normal | 7 (17.9) | 1 (7.1) | 3 (30) | 3 (42.8) | 0 (0) | 0 (0) | 0 (0) |
| Other | | | | | | | |
| Polyps | 3 (7.6) | 2 (14.2) | 1 (10) | 0 (0) | 0 (0) | 0 (0) | 0 (0) |
| Histologic findings | | | | | | | |
| Acute inflammation | 18 (46.1) | 6 (42.8) | 4 (40) | 2 (28.5) | 4 (66.6) | 0 (0) | 0 (0) |
| Chronic inflammation | 10 (25.6) | 4 (28.4) | 2 (20) | 3 (42.8) | 1 (16.6) | 0 (0) | 0 (0) |
| Microscopic colitis | 3 (7.6) | 2 (14.2) | 0 (0) | 0 (0) | 1 (16.6) | 0 (0) | 0 (0) |
| Normal | 8 (20.5) | 2 (14.2) | 4 (40) | 2 (28.5) | 0 (0) | 0 (0) | 0 (0) |
| Treatment of IMC | | | | | | | |
| Corticosteroids only | 16 (41.0) | 7 (50.0) | 2 (20) | 4 (57.1) | 2 (33.3) | 1 (100) | 0 (0) |
| Corticosteroids plus infliximab only | 8 (20.5) | 1 (7.1) | 5 (50) | 0 (0) | 2 (33.3) | 0 (0) | 0 (0) |
| Corticosteroids plus vedolizumab only | 7 (17.9) | 0 (0) | 1 (10) | 3 (42.8) | 2 (33.3) | 0 (0) | 1 (100) |
| Multiple treatment lines | 8 (20.5) | 6 (42.8) | 2 (20) | 0 (0) | 0 (0) | 0 (0) | 0 (0) |
| **At the time of follow-up endoscopy** | | | | | | | |
| Endoscopic findings | | | | | | | |
| Inflammation | | | | | | | |

| | | | | | | | |
|---|---|---|---|---|---|---|---|
| Erythema, erosions, vascular congestion and inflammation | 16 (41) | 8 (57.1) | 4 (40) | 2 (28.5) | 2 (33.3) | 0 (0) | 0 (0) |
| Normal | 23 (58.9) | 6 (42.8) | 6 (60) | 5 (71.4) | 4 (66.6) | 1 (100) | 1 (100) |
| Other | | | | | | | |
| New polyps | 20 (51.2) | 7 (50) | 5 (50) | 4 (57.1) | 3 (50) | 0 (0) | 1 (100) |
| Stricture | 0 (0) | 0 (0) | 0 (0) | 0 (0) | 0 (0) | 0 (0) | 0 (0) |
| Perforation | 0 (0) | 0 (0) | 0 (0) | 0 (0) | 0 (0) | 0 (0) | 0 (0) |
| Histologic findings | | | | | | | |
| Inflammatory findings | | | | | | | |
| Inflammation | 19 (48.7) | 8 (57.1) | 4 (40) | 3 (42.8) | 4 (66.6) | 0 (0) | 0 (0) |
| Normal histologic findings | 20 (51.2) | 6 (42.8) | 6 (60) | 4 (57.1) | 2 (33.3) | 1 (100) | 1 (100) |
| Other | | | | | | | |
| Adenoma polyps | 13 (33.3) | 6 (42.8) | 3 (30) | 2 (28.5) | 1 (16.6) | 0 (0) | 1 (100) |

Abbreviations: CTCAE v5, Common Terminology Criteria for Adverse Events version 5; ICI, immune checkpoint inhibitor; IMC, immune-mediated colitis; IQR, interquartile range; TNF, tumor necrosis factor; FMT, fecal microbiota transplantation; GU, genitourinary; GI, gastrointestinal.

| **Supplemental Table 2.** Characteristics of patients with and without adenoma, N=39 | | | |
|---|---|---|---|
| **Characteristic** | **Adenoma polyps* No. (%) N=13** | **No adenoma polyps No. (%) N=26** | ***p*** |
| **Baseline characteristics** | | | |
| Age, years, median (IQR) | 66 (60-70) | 62 (48-68) | 0.268 |
| Sex = male | 11 (84.6) | 21 (80.7) | 1.000 |
| Race = white | 11 (84.6) | 24 (92.2) | 0.589 |
| Cancer type | | | 0.943 |
| Melanoma | 6 (46.1) | 8 (30.7) | |
| Genitourinary | 3 (23) | 7 (26.9) | |
| Gastrointestinal | 3 (23.1) | 4 (15.3) | |
| Lung/head/neck | 1 (7.6) | 5 (19.2) | |
| Hematological | 0 (0) | 1 (3.8) | |
| Renal | 0 (0) | 1 (3.8) | |
| Type of ICI | | | 0.180 |
| Anti–CTLA-4 monotherapy | 0 (0) | 2 (7.6) | |
| Anti–PD-1/L1 monotherapy | 0 (0) | 5 (19.2) | |
| Combination anti–CTLA-4 and anti–PD-1/L1 | 13 (100) | 19 (73) | |
| Previous colonoscopy screening before colitis | 3 (23.1) | 3 (11.5) | 0.380 |
| Duration of previous colonoscopy before colitis to colitis onset, months, median (IQR), n=6 | 7.0 (6.1-16.4) | 9.7 (4.8-35.4) | 1.000 |
| Personal history of colon polyps before colitis, n=6 | 3 (100) | 3 (100) | -- |
| Characteristics of index colitis | | | |
| Polyps on initial endoscopic evaluation for colitis | 2 (15.4) | 1 (3.8) | 0.253 |
| Grades of preparation on initial endoscopy, n=30‡ | | | 1.000 |
| Adequate, good, or excellent preparation | 6 (60) | 13 (60) | |

| | | | |
|---|---|---|---|
| Fair or poor preparation | 4 (40) | 7 (30) | |
| Endoscopic findings of initial colitis event | | | 0.703 |
| Ulcers | 0 (0) | 2 (7.6) | |
| Non-ulcer inflammation | 10 (76.9) | 20 (76.9) | |
| Normal | 3 (23.1) | 4 (15.3) | |
| Histologic findings of initial colitis event | | | 0.637 |
| Acute | 5 (38.4) | 13 (50) | |
| Chronic | 3 (23.1) | 7 (26.9) | |
| Normal | 3 (23.1) | 5 (19.2) | |
| Microscopic colitis | 2 (15.3) | 1 (3.8) | |
| Treatment of colitis | | | 0.193 |
| Corticosteroids alone | 7 (53.8) | 10 (38.4) | |
| Multiple treatment lines† | 6 (46.1) | 16 (53.8) | |
| At the time of follow-up endoscopy | | | |
| Grade of preparation on follow-up endoscopy | | | 1.000 |
| Adequate, good, or excellent preparation, n=30§ | 7 (70) | 17 (85) | |
| Fair or poor preparation | 3 (30) | 3 (15) | |
| Active inflammation on follow-up endoscopy | 3 (30) | 13 (50) | 0.176 |
| Active inflammation on follow-up histology | 10 (76.9) | 9 (34.6) | 0.380 |
| Duration from colitis diagnosis to last follow-up endoscopy, months, median (IQR) | 14.8 (8.9-30.5) | 4.11 (2.4-15.2) | 0.004* |
| Duration from colitis diagnosis to last follow-up in months, median (IQR) | 13.6 (11.2-17.15) | 15.5 (7.8-41.2) | 0.780 |

Abbreviations: CTLA-4, cytotoxic T lymphocyte antigen 4; FMT, fecal microbiota transplant; ICI, immune checkpoint inhibitor; IQR, interquartile range; PD-1/PD-L1, programmed cell death 1/programmed death ligand 1.

7 patients (18%) were females.
3 patients (7.6%) were Hispanic and 1 patient (2.5%) African American.

[*]11 patients had tubular adenomas, 2 had tubulovillous adenomas. 1 patient had polyps <5 mm in size, and 11 had polyps >5 mm.

[†]Other add-on biologic treatment included the following; infliximab only: 2 (15.3%) patients in the adenoma group and 6 (23%) patients in the non-adenoma group, vedolizumab only: 3 (23%) patients in the adenoma group and 6 (23%) patients in the non-adenoma group, both infliximab and vedolizumab: 1 (7.6%) patients in the adenoma group and 3 (11.5%) patients in the non-adenoma group, ustekinumab add-on: 1 (3.8%) patient in the non-adenoma group. 4 patients in the non-adenoma group received FMT (1 FMT + corticosteroids, 2 FMT + vedolizumab, 1 FMT + ustekinumab).

‡30 patients had described the grade of bowel preparation on initial endoscopy, the remaining 9 patients were missing this feature.

§30 patients had described the grade of bowel preparation on follow-up endoscopy, the remaining 9 patients were missing this feature.
